# Quantum Weak Values and the “Which Way?” Question

**DOI:** 10.3390/e27030259

**Published:** 2025-03-01

**Authors:** Anton Uranga, Elena Akhmatskaya, Dmitri Sokolovski

**Affiliations:** 1 Basque Center for Applied Mathematics (BCAM), Alameda de Mazarredo 14, 48009 Bilbao, Spain; 2 Departmento de Química-Física, Universidad del País Vasco, UPV/EHU, 48940 Leioa, Spain; 3 IKERBASQUE, Basque Foundation for Science, Plaza Euskadi 5, 48009 Bilbao, Spain; 4 EHU Quantum Center, Universidad del País Vasco, UPV/EHU, 48940 Leioa, Spain

**Keywords:** quantum foundations, weak values, quantum optics, Feynman paths

## Abstract

The Uncertainty Principle forbids one to determine which of the two paths a quantum system has travelled, unless interference between the alternatives had been destroyed by a measuring device, e.g., by a pointer. One can try to weaken the coupling between the device and the system in order to avoid the veto. We demonstrate, however, that a weak pointer is at the same time an inaccurate one, and the information about the path taken by the system in each individual trial is inevitably lost. We show also that a similar problem occurs if a classical system is monitored by an inaccurate quantum meter. In both cases, one can still determine some characteristic of the corresponding statistical ensemble, a relation between path probabilities in the classical case, and a relation between the probability amplitudes if a quantum system is involved.

## 1. Introduction

There is a well-known difficulty with determining the path taken by a quantum system capable of reaching a known final state via several alternative routes. According to the Uncertainty Principle [1], such a determination is possible only if an additional measuring device destroys interference between the alternatives. However, the device inevitably perturbs the system’s motion, and alters the likelihood of its arrival at the desired final state. The knowledge of the system’s past must, therefore, be incompatible with keeping the probability of a successful post-selection intact.

A suitable measuring device can be a pointer [2], designed to move only if the system travels the chosen path so that finding it displaced at the end of experiment could constitute a proof of the system’s past. For practical aspects of quantum measurements, see, for example [3,4]. A somewhat naive way around the Uncertainty Principle may be the use of a pointer coupled to the system only weakly, thus leaving interference between the paths almost intact. Perhaps the small change in the pointer’s final state caused by the weak interaction could provide “which path?” (“which way?”) information previously deemed to be unavailable.

This change can be expressed in terms of a “weak value” (WV) [5] of a quantity $\pi_{j}$, which takes a unit value for the path of interest, say, the path number *j*, and vanishes otherwise. The complex valued WV is conveniently defined as the ratio ${\langle \pi_{j}\rangle }_{Weak}\equiv A_{j}/\sum_{i}A_{i}$, where $A_{i}$ is the probability amplitude that quantum theory ascribes to the *i*-th path available to the system. For a quantity *B* whose value on the *i*-th path is $B_{i}$, the WV is $B_{W}=\sum_{i}B_{i}{\langle \pi_{i}\rangle }_{W}$. The quantity ${\langle \pi_{j}\rangle }_{W}$, always known to the theoretician, can also be measured by the practitioner. (No surprise here; the response of a quantum system to a small perturbation is usually expressed in terms of probability amplitudes rather than probabilities.)

The problem with the just described “weak measurements” is to ascribe a physical meaning to a “weak value” which is, after all, a particular combination of the system’s amplitudes. Quantum theory provides only one firm rule: an absolute square $|A_{j}{|}^{2}$ yields the relative frequency with which the system travels the *j*-th path, should the interference between the paths be destroyed by a measuring device. But can there be more rules? For example, what is $A_{j}/\sum_{i}A_{i}$? The mean shift of the “weak” pointer. Yes, but what does it say about the system? The number one obtains dividing $A_{j}$ by the sum of the amplitudes leads to the same final state? Certainly, the theoretician can figure out this number on the back of an envelope. But what, we insist, does it tell about the system? The question is one of principle: would the knowledge of a WV reveal anything previously unknown about the route by which the system reaches its final destination?

The idea is not new, and was applied, for example, to an optical realisation of a three-path problem [6,7]. The conclusion that the photons can be found in a part of the setup they can neither enter nor leave, and must therefore have discontinuous trajectories, was subsequently criticised by a number of authors for both technical and more fundamental reasons [8,9,10,11,12,13,14,15,16,17,18,19,20,21,22,23,24]. A similar treatment of a four-path “quantum Cheshire cat” [25,26] model suggests the possibility of separating a system from its property, to wit, electrons detached from their charges, and an atom’s internal energy “disembodied” from the atom itself. (For further discussion of the model, the reader is referred to [27]). The case for a quantum particle (or, at least, of some of its “properties”) being in several interfering pathways at the same time was recently made in [28].

Here, our more modest aim is to analyse, in some detail, the validity of the approach in the case of the simplest “double-slit” (two-path) problem.

The rest of the paper is organised as follows. Section 2 briefly describes the well-known quantum double-slit experiment. A classical analogue of the problem is studied in Section 3, Section 4, Section 5 and Section 6. A simple two-way quantum problem is analysed in Section 7, Section 8, Section 9, Section 10 and Section 11. Section 12 contains our conclusions.

## 2. Quantum “Which Way?” Problem

**Figure 1:**
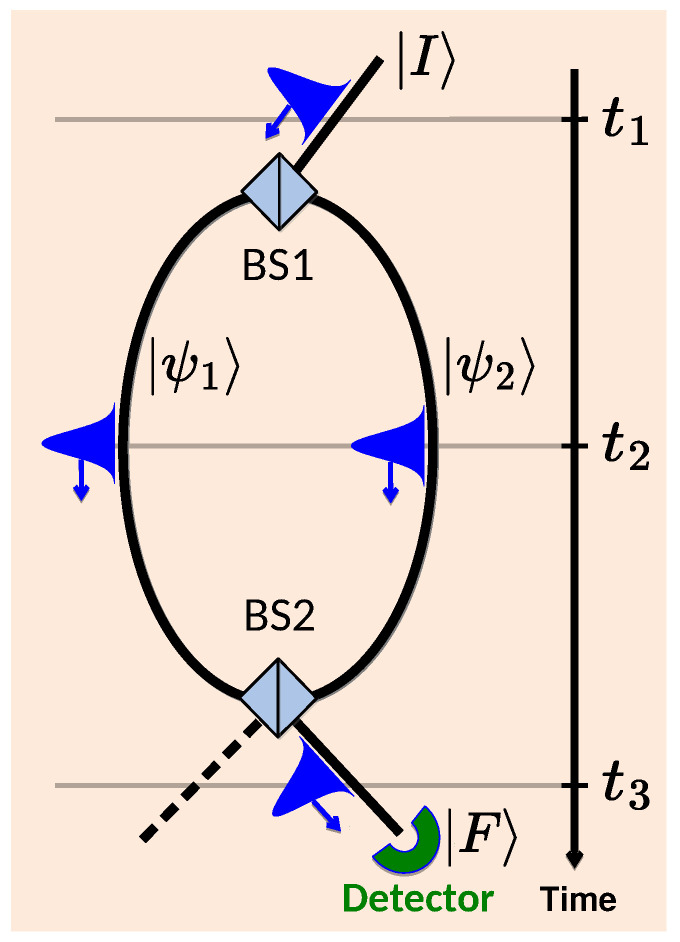
An optical realisation of a “double-slit” experiment. At time $t_{1}$, a photon’s wave packet |I〉 is split into two at the first beam splitter (BS1). Its parts $|\psi_{1}\rangle$ and $|\psi_{2}\rangle$ travel both optic fibres $t_{2}$ and are recombined into |F〉 after the second BS2 $t_{3}$. The two observed events are the initial preparation and the final detection of the photon. Where was the photon between these two events?

One of the unanswered questions in quantum theory, indeed its “only mystery” [29], concerns the behaviour of a quantum particle in a double-slit experiment shown in Figure 1. The orthodox [1] view is as follows. With only two observable events, preparation and final detection, it is impossible to claim that the particle has gone via one of the slits (paths) and not the other. This is because the rate of detection by the detector in Figure 1 may increase if one of the paths is blocked [1].

Neither is it possible to claim that both paths were travelled at the same time since an additional inspection never finds only a fraction of a photon in one of the paths [29].

However, such an inspection destroys the interference between the paths, and alters the probability of detection. The problem is summarised in the Uncertainty Principle [29]: “It is impossible to design any apparatus whatsoever to determine through which hole the particle passes that will not at the same time disturb the particle enough to destroy the interference”.

A brief digression into Bohmian theory [30] is in order. One can treat the flow lines of a probability density (Bohmian trajectories), obtained from a Schrödinger wave function, as a quantum particle’s trajectories. If so, in the double-slit case, one finds a single trajectory leading to a given point on the screen and passing through one of the slits. However, a contradiction with the Uncertainty Principle stated above is only specious. Bohmian mechanics reproduces all results of the conventional theory, and the problem of receiving more particles with one slit closed still defies a classical-like explanation. The comfort of “knowing” where the particle was at all times is bought at the price of introducing quantum potential with unusual and potentially non-local properties. Bohmian trajectories can be evaluated by a theoretician and reconstructed from the “weak values” measured by the experimenter [31,32,33,34,35]. The question is how to use or interpret these trajectories once they have been obtained, in one way or another. The consensus appears to be moving away from the original interpretation. Thus, Hiley and Van Reeth suggest [36] that “the flow lines *…* are not the trajectories of single atoms but an average momentum flow”. Furthermore, Flack and Hiley [37] relate them to Feynman paths (more relevant for our analysis). Similarly, the authors of [32,34] refrain from identifying the average momentum flow lines with individual photon trajectories. Another reason why the Bohmian perspective is of little interest for the present work is because, below, we will limit ourselves to the study of systems in two-dimensional Hilbert spaces, where the application of the method is at best problematic.

**Figure 2:**
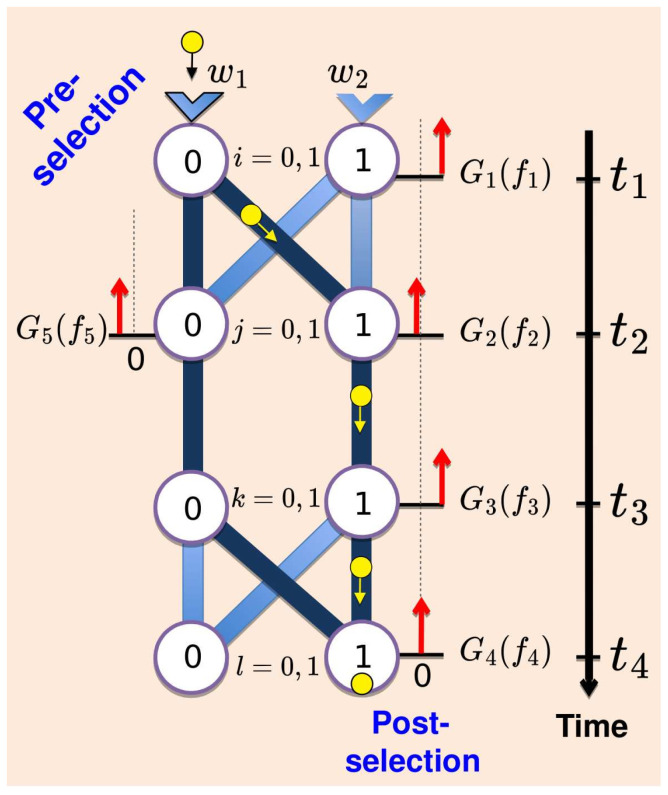
A classical two-state system can reach a final state by taking one of the the eight paths with a probability given by Equation (1). The system can be monitored with the help of pointers (red arrows) which move if the system is detected [cf. Equation (2)]. Also shown (in dark blue) is the two-way problem of Section 4.

We return, therefore, to the possibility of finding a way around the Uncertainty Principle by perturbing the measured system only slightly. One such approach, first proposed by Vaidman in Ref. [6], suggests the following. If two von Neumann pointers [2], set up to measure projectors on the paths (e.g., on the states $|\psi_{1}\rangle$ and $|\psi_{2}\rangle$ in Figure 1), are coupled to the particle only weakly, interference between the paths can be preserved. (One can perform non-perturbing (weak) measurements using von Neumann pointers, either by making coupling to the observed system weak or, equivalently, by making the pointer’s initial position uncertain as we consider.) If, in addition, both pointers are found to “have moved”, albeit on average, the “weak traces” [6] left by the particle will reveal its presence in both paths at the same time. The idea appears to contradict the Uncertainty Principle and, for this reason, deserves our attention. We start the investigation by looking first at inaccurate pointers designed to monitor a classical stochastic system in Figure 2.

## 3. Consecutive Measurements of a Classical System

Our simple classical model is as follows. (We ask for the reader’s patience. The quantum case will be discussed shortly.) A system (one can think of a little ball rolling down a network of connected tubes shown in Figure 2) is introduced into one of the two inputs at $t=t_{1}$, with a probability $w_{i}$, i=0,1. It then passes through states *j* and *k*, where j,k=0,1 at the times $t_{2}$, and $t_{3}$, respectively. The experiment is finished when the system is collected in a state *l*, l=0,1 at $t=t_{4}$. From each state *i*, the system is directed to one of the states *j* with a probability p(j←i), similarly from *j* to *k*, and finally from *k* to *l*. There are altogether eight paths {l←k←j←i}, each travelled with a probability

(1) $$\begin{array}{cc} \; & P(l\leftarrow k\leftarrow j\leftarrow i)=p(l\leftarrow k)p(k\leftarrow j)p(j\leftarrow i),\\ \; & p\left(k\leftarrow j\right)=\delta_{jk}, \end{array}$$

where $\delta_{jk}$ is the Kronecker delta. (The choice of this design will become clear shortly.)

We make the following assumptions.

1. Alice, the experimenter, knows the path probabilities in Equation (1) but not the input values $w_{i}$.
2. She cannot observe the system directly, and relies on the readings of pointers with positions $f_{n}$, n=1,2,…,5, installed at different locations as shown in Figure 2. If the system passes through a location, the corresponding pointer is displaced by a unit length, $f_{n}\to f_{n}+1$; otherwise, it is left intact.
3. The pointers are, in general, inaccurate since their initial positions are distributed around zero with probabilities $G_{n}\left(f_{n}\right)$ (see Figure 2). Their final positions are, however, determined precisely. We will consider the distributions $G_{n}$ to be Gaussians of widths $\Delta f_{n}$:
  (2) $$\begin{array}{cc} \; & G_{n}\left(f_{n}\right)=\sqrt{\frac{1}{\pi {\left(\Delta f_{n}\right)}^{2}}}\exp\left(-\frac{f_{n}^{2}}{{\left(\Delta f_{n}\right)}^{2}}\right),\\ \; & \int G_{n}\left(f_{n}\right)df_{n}=1,\;G_{n}\left(f_{n}\right)\underset{\Delta f_{n}\to 0}{\to }\delta \left(f_{n}\right). \end{array}$$

The experiment ends just after $t=t_{4}$, when Alice’s observed outcomes are the five numbers $f_{n}$, n=1,⋯,5. These are distributed with a probability density

(3) $$\begin{array}{cc} \rho \left(f_{1},f_{2},f_{3},f_{4},f_{5}\right)=\sum_{i,j,k,l=0,1} & w_{i}P\left(l\leftarrow k\leftarrow j\leftarrow i\right)\times \\ \; & G_{1}\left(f_{1}-i\right)G_{2}\left(f_{2}-j\right)G_{3}\left(f_{3}-k\right)G_{4}\left(f_{4}-l\right)G_{5}\left(f_{5}+j-1\right). \end{array}$$

Equation (3) is not particularly useful since $w_{i}$ are unknown. However, by making the first pointer accurate, $\Delta f_{1}\to 0$, $G_{1}\left(f_{1}\right)\to \delta \left(f_{1}-i\right)$ where δ(x) is the Dirac delta, and she is able to *pre-select* those cases, where, say, $f_{1}=0$, and collect only the corresponding statistics. Now the (properly normalised) distribution of the remaining four readings does not depend on $w_{i}$,

(4) $$\begin{array}{c} \rho_{0}\left(f_{2},f_{3},f_{4},f_{5}\right)=\sum_{j,k,l=0,1}P\left(l\leftarrow k\leftarrow j\leftarrow 0\right)G_{2}\left(f_{2}-j\right)G_{3}\left(f_{3}-k\right)G_{4}\left(f_{4}-l\right)G_{5}\left(f_{5}+j-1\right), \end{array}$$

and Alice has a complete description of the pre-selected ensemble.

Alice can also *post-select* the system by selecting, for example, the cases where it ends in a state 1 at $t=t_{4}$. With $G_{4}\left(f_{4}\right)\to \delta \left(f_{4}-1\right)$, the remaining random variables $f_{2}$, $f_{3}$, and $f_{5}$ are distributed according to [cf. Figure 2 and Equation (1)]

(5) $$\begin{array}{c} \rho_{1\leftarrow 0}\left(f_{2},f_{3},f_{5}\right)=\frac{\sum_{j=0,1}P_{j}G_{2}\left(f_{2}-j\right)G_{3}\left(f_{3}-j\right)G_{5}\left(f_{5}+j-1\right)}{P_{0}+P_{1}}, \end{array}$$

where we introduce a shorthand

(6) $$\begin{array}{c} P_{j}\equiv P\left(1\leftarrow j\leftarrow j\leftarrow 0\right),\;j=0,1. \end{array}$$

Equation (4) suggests a simple, yet useful, general criterion.

- Alice can determine the system’s past location *only* when she obtains a pointer’s reading whose likelihood depends *only* on the probabilities of the system’s paths passing through that location.

For example, at least three accurate readings ($f_{1}$, $f_{4}$ and one of $f_{2}$,$f_{3}$ or $f_{5}$,) are needed if Alice is to know which of the eight paths shown in Figure 2 the system has travelled during each trial. With $G_{n}\left(f_{n}\right)=\delta \left(f_{n}\right)$ for n=1,2,4, a trial can yield, for example, the values $f_{1}=0$, $f_{2}=1$, and $f_{4}=1$. The likelihood of these outcomes is given by the probability P(1←1←1←0) in Equation (1), and Alice can be certain that the route {1←1←1←0} has indeed been travelled.

## 4. A Classical “Two-Way Problem”

Consider next a pre- and post-selected ensemble with two routes connecting the states 0 at $t=t_{1}$ and 1 at $t=t_{4}$ (shown in Figure 2 in dark blue). As a function of the second pointer’s accuracy, $\Delta f_{2}$, the distribution of its readings (5) changes from a bimodal, when the pointer is accurate

(7) $$\begin{array}{cc} \rho_{1\leftarrow 0}\left(f_{2}\right)\equiv \int \rho_{1\leftarrow 0}\left(f_{2},f_{3},f_{5}\right)df_{3}df_{5}= & \frac{P_{0}G_{2}\left(f_{2}\right)+P_{1}G_{2}\left(f_{2}-1\right)}{P_{0}+P_{1}}\\ & \;\underset{\Delta f_{2}\to 0}{\to }\frac{P_{0}\delta \left(f_{2}\right)+P_{1}\delta \left(f_{2}-1\right)}{P_{0}+P_{1}} \end{array}$$

to the original broad Gaussian for an inaccurate pointer,

(8) $$\begin{array}{c} \rho_{1\leftarrow 0}\left(f_{2}\right)\underset{\Delta f_{2}\to \infty }{\to }\;\;G_{2}\left(f_{2}-z\right),\;\;\; \end{array}$$

displaced as a whole by

(9) $$\begin{array}{c} z=\frac{P_{1}}{P_{0}+P_{1}}. \end{array}$$

**Figure A1:**
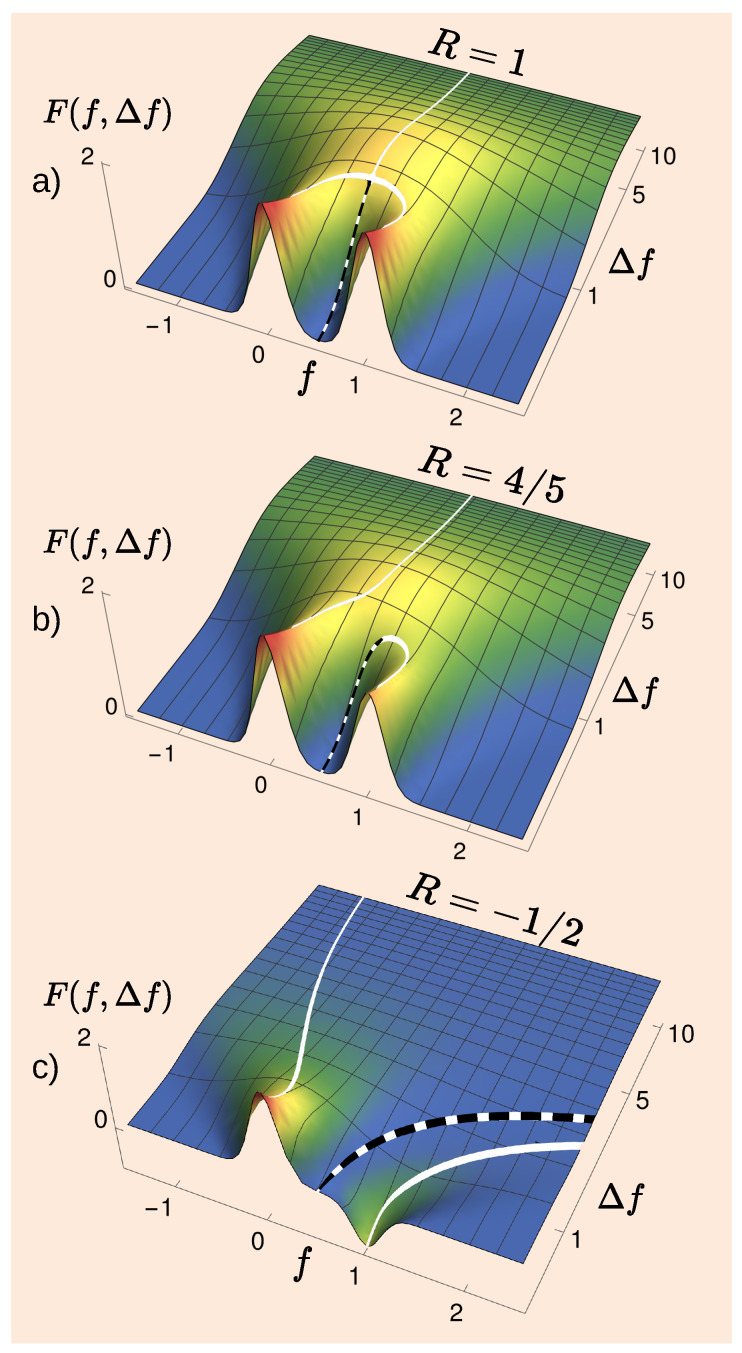
Maxima (solid white) and minima (dashed) of F(f) vs. *f* and Δf (a.u.). (**a**) Pitchfork bifurcation for R=1, (**b**) the same but for R=4/5, and (**c**) the same but for a negative R=−1/2. Note that in this case, one has an “anomalous” value z=−1.

Equation (8) reflects a known property of Gaussians, to our knowledge, first explored in [38], and discussed in detail in Appendix A. The transformation of two peaks (7) into a single maximum (8) is best described by the catastrophe theory [39]. For example, for $P_{0}=P_{1}$, a pitchfork bifurcation converts two maxima and a minimum into a single maximum for $\Delta f_{2}=\sqrt{2}$ (see Figure A1a of Appendix B).

With a sufficiently accurate pointer $\Delta f_{2}\ll 1$, a reading always lies close to 0 or 1, and in every trial, Alice knows the path followed by the system.

With a highly inaccurate pointer $\Delta f_{2}\gg 1$, not a single reading $f_{2}$ can be attributed to one path in preference to the other, and the route by which the system arrived at its final state is never known (see Appendix C). Indeed, for $P_{0}=P_{1}$, even the most probable outcome $f_{2}=1/2$ is equally likely to occur if the system takes path {1←0←0←0}, or {1←1←1←0},

(10) $$\begin{array}{c} \rho \left(f_{2}=\frac{1}{2}\right)=\frac{1}{2}\left[G_{2}\left(\frac{1}{2}\right)+G_{2}\left(-\frac{1}{2}\right)\right], \end{array}$$

and the “which way?” information is clearly lost.

Still, something can be learned about a pre- and post-selected classical ensemble, even without knowing the path taken by the system. Having performed many trials, Alice can evaluate an average reading,

(11) $$\begin{array}{c} \langle f_{2}\rangle \equiv \int f_{2}\rho_{1\leftarrow 0}\left(f_{2}\right)df_{2}=z. \end{array}$$

The quantity *z* in Equations (9) and (11) is the relative (i.e., renormalised to a unit sum) probability of travelling the path {1←1←1←0}, and is independent of $\Delta f_{2}$. Thus, by using an inaccurate pointer, Alice can still estimate certain parameters of her statistical ensemble.

## 5. Two Inaccurate Classical Pointers and a Wrong Conclusion

**Figure 3:**
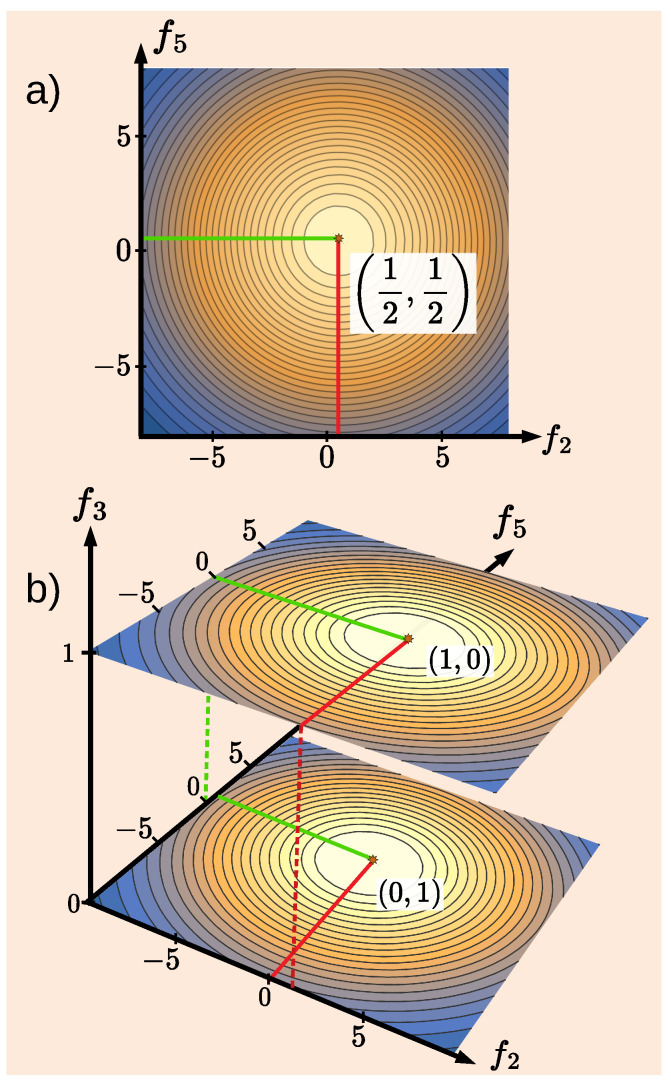
(**a**) Distribution of the readings $\rho_{1\leftarrow 0}\left(f_{2},f_{5}\right)$ of two inaccurate pointers with $P_{0}=P_{1}$ and $\Delta f_{2}=\Delta f_{5}=10$, monitoring a classical system at $t=t_{2}$ [cf. Equation (12)]; (**b**) the distribution $\rho_{1\leftarrow 0}\left(f_{2},f_{3},f_{5}\right)$ in Equation (14) with an accurate pointer, $\Delta f_{3}\to 0$, added at $t=t_{3}$. Note that the integration of the distribution in (**b**) over $df_{3}$ recovers the distribution shown in (**a**).

A word of caution should be added against an attempt to recover the “which way?” information with the help of Equation (11). For two equally inaccurate pointers, $\Delta f_{2}=\Delta f_{5}\equiv \Delta f\gg 1$ [cf. Figure 2], the distribution of the readings tends to a single Gaussian shown in Figure 3a (see also Appendix A),

(12) $$\begin{array}{c} \rho_{1\leftarrow 0}\left(f_{2},f_{5}\right)=\frac{P_{0}G_{2}\left(f_{2}\right)G_{5}\left(f_{5}-1\right)+P_{1}G_{2}\left(f_{2}-1\right)G_{5}\left(f_{5}\right)}{P_{0}+P_{1}}\underset{\Delta f\to \infty }{\to }\;G_{2}\left(f_{2}-z_{2}\right)G_{5}\left(f_{5}-z_{5}\right), \end{array}$$

where

(13) $$\begin{array}{c} z_{2}=\frac{P_{1}}{P_{0}+P_{1}},\;z_{5}=\frac{P_{0}}{P_{0}+P_{1}}=1-z_{2}. \end{array}$$

It may seem that (the reader can already see where we are going with this) the following hold:

(i) Each pointer in Equation (12) “moves” only when the system is in its path.
(ii) Equation (12) suggests that both pointers have moved (albeit on average).
(iii) Hence, the system must be travelling both paths at the same time.

To check if this is the case, Alice can add an accurate pointer ($f_{3}$) acting at $t=t_{3}$ (see Figure 2). If parts of the system were in both places at $t_{2}$, the same must be true at $t_{3}$ since Alice makes sure that no pathway connects the points j=0 and k=1. The accurate pointer should, therefore, always find only a part of the system. Needless to say, this is not what happens. With an additional accurate pointer in place, three-dimensional distribution (5) becomes bimodal, again (see Figure 3b)

(14) $$\begin{array}{c} \rho_{1\leftarrow 0}\left(f_{2},f_{3},f_{5}\right)={\left(P_{0}+P_{1}\right)}^{-1}\left[P_{0}G_{2}\left(f_{2}\right)G_{5}\left(f_{5}-1\right)\delta \left(f_{3}\right)+P_{1}G_{2}\left(f_{2}-1\right)G_{5}\left(f_{5}\right)\delta \left(f_{3}-1\right)\right]. \end{array}$$

An inspection of statistics collected separately for $f_{3}=0$ or $f_{3}=1$ shows that at $t=t_{2}$, only one of the two pointers moves in any given trial. Contour plots of the densities in Equations (12) and (14) are shown in Figure 3a,b, respectively. The fallacy (i)–(iii), evident in our classical example, will become less obvious in the quantum case we will study after a brief digression.

## 6. Classical “Hidden Variables”

Before considering the quantum case, it may be instructive to add a fourth assumption to the list of Section 3.

4. In Alice’s world, all pointers have the property that an accurate detection inevitably perturbs the system’s evolution.

For example, whenever the pointer $f_{3}$ moves, the probabilities p(l←1) in Equation (1) are reset to $p^{\prime }\left(l\leftarrow 1,\Delta f_{3}\right)$. Thus, $P_{1}$ changes to $P_{1}^{\prime }(\Delta f_{3}$), while $P_{0}$ remains the same. The change may be the greater the smaller $\Delta f_{3}$ is and $P_{1}^{\prime }\left(\Delta f_{3}\to \infty \right)=P_{1}$. Now the system, accurately observed in the state 1 at $t_{3}$ arrives in state 1 at $t=t_{4}$, say, less frequently than it would with no pointer ($f_{3}$) in place, $P_{0}+P_{1}^{\prime }\left(\Delta f_{3}\to 0\right)<P_{0}+P_{1}$. So, where was the *unobserved* system at $t=t_{3}$?

Empirically, the question has no answer. To ensure the arrival rate is unchanged by observation, Alice can only use an inaccurate pointer, $\Delta f_{3}\;\gg 1$, which yields no “which way?” information. Performing many trials, she can, however, measure both the probability of arriving in 1 at $t=t_{4}$, $W_{1}\left(t_{4}\right)=P_{0}+P_{1}^{\prime }\left(\Delta f_{3}\gg 1\right)\approx P_{0}+P_{1}$ and the value of $z=P_{1}^{\prime }\left(\Delta f_{3}\gg 1\right)/\left[P_{0}+P_{1}^{\prime }\left(\Delta f_{3}\gg 1\right)\right]\approx P_{1}/\left[P_{0}+P_{1}\right]$ [cf. Equations (8) and (9)]. She can then evaluate unperturbed path probabilities,

(15) $$\begin{array}{c} P_{1}\approx zW_{1}\left(t_{4}\right),\;P_{0}\approx \left(1-z\right)W_{1}\left(t_{4}\right). \end{array}$$

Having observed that $P_{0}$ and $P_{1}$ are both positive, and do not exceed unity, Alice may reason about what happens to the unobserved system in the following manner. The available empirical data are consistent with the system, always following one of the two paths with probabilities in Equation (15). However, with the available instruments, it is not possible to verify this conclusion experimentally.

This is as close as we can get to the quantum case using a classical toy model. We consider the quantum case next.

## 7. Consecutive Measurements of a Qubit

**Figure 4:**
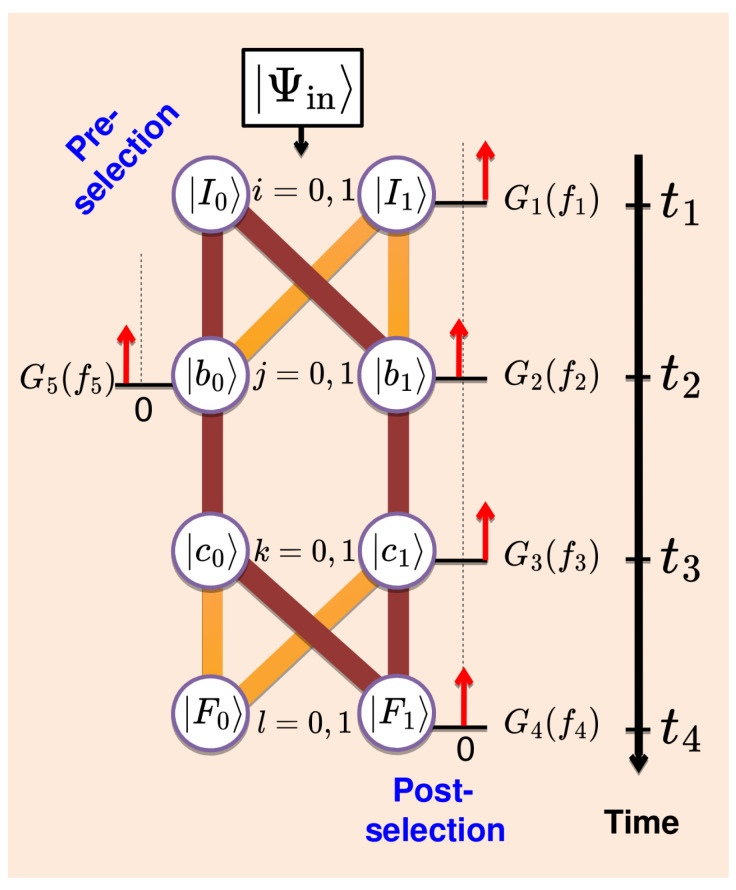
A two-level quantum system can reach final states $|F_{l}\rangle$, l=0,1, via eight virtual paths whose probability amplitudes are given by Equation (16). The system is monitored by means of von Neumann pointers (red arrows), set to measure projectors ${\hat{\pi }}_{n}$ [cf. Equation (17)]. Also shown (in maroon) is the “double-slit problem” of Section 8.

A quantum analogue of the classical model just discussed is shown in Figure 4. An experiment in which Alice monitors the evolution of a two-level quantum system (qubit) with a Hamiltonian ${\hat{H}}^{s}$ by means of five von Neumann pointers begins at $t=t_{1}$ and ends at $t=t_{4}$. With no transitions between the states $|b_{0(1)}\rangle$ and $|c_{1(0)}\rangle$, there are altogether eight virtual (Feynman) paths which connect the initial and final states. Just before $t_{1}$, the qubit may be thought to be in some state $|\Psi_{\mathrm{in}}\rangle$, and the eight path amplitudes are given by (i,j,k,l=0,1) [cf. Figure 4]

(16) $$\begin{array}{c} A^{s}\left(F_{l}\leftarrow c_{j}\leftarrow b_{j}\leftarrow I_{i}\right)=a\left(F_{l}\leftarrow c_{j}\right)a\left(c_{j}\leftarrow b_{j}\right)a\left(b_{j}\leftarrow I_{i}\right), \end{array}$$

where $a\left(b_{j}\leftarrow I_{i}\right)=\langle b_{j}|{\hat{U}}^{s}\left(t_{2}-t_{1}\right)|I_{i}\rangle$, etc., and ${\hat{U}}^{s}\left(t\right)\equiv \exp\left(-i{\hat{H}}^{s}t\right)$ is the qubit’s own evolution operator.

We note the following.

1. Alice the experimenter knows the path amplitudes in Equation (16) but not the system’s input state $|\Psi_{\mathrm{in}}\rangle$. (If she did, the experiment would begin earlier, at the time $|\Psi_{\mathrm{in}}\rangle$ was first determined.)
2. Alice cannot look at the system directly and has access only to von Neumann pointers [2], with positions $f_{n}$, and momenta $\lambda_{n}$, n=1,…,5 (see Figure 4). The pointers are briefly coupled to the system at $t=t_{n}$, ($t_{5}\equiv t_{2}$), via
  (17) $$\begin{array}{c} {\hat{H}}_{n}^{\mathrm{int}}=-i\partial_{f_{n}}{\hat{\pi }}_{n}\delta \left(t-t_{n}\right), \end{array}$$
  where
  (18) $$\begin{array}{c} {\hat{\pi }}_{1}=|I_{1}\rangle \langle I_{1}|,\;\;{\hat{\pi }}_{2}=|b_{1}\rangle \langle b_{1}|,\;\;{\hat{\pi }}_{3}=|c_{1}\rangle \langle c_{1}|,\;\;{\hat{\pi }}_{4}=|F_{1}\rangle \langle F_{1}|,\;\;{\hat{\pi }}_{5}=|b_{0}\rangle \langle b_{0}| \end{array}$$
  and have no own dynamics.
3. The pointers, initially in states $|G_{n}\rangle$, are inaccurate, with initial positions distributed around zero with probability amplitudes $G_{n}\left(f_{n}\right)\equiv \langle f_{n}|G_{n}\rangle$. We consider Gaussian pointers,
  (19) $$\begin{array}{cc} \; & G_{n}\left(f_{n}\right)={\left(\frac{2}{\pi {\left(\Delta f_{n}\right)}^{2}}\right)}^{1/4}\exp\left(-\frac{f_{n}^{2}}{{\left(\Delta f_{n}\right)}^{2}}\right),\\ \; & \int G_{n}^{2}\left(f_{n}\right)df_{n}=1,\;\;G_{n}^{2}\left(f_{n}\right)\underset{\Delta f_{n}\to 0}{\to }\delta \left(f_{n}\right). \end{array}$$
4. A pointer perturbs the qubit’s evolution, except in the limit $\Delta f_{n}\to \infty$. Indeed, replacing $f_{n}$ with $f_{n}^{\prime }=f_{n}/\Delta f_{n}$ changes ${\hat{H}}^{\mathrm{int}}$ in Equation (17) to ${\hat{H}}^{{\mathrm{int}}^{\prime }}={\hat{H}}^{\mathrm{int}}/\Delta f_{n}$, and a highly inaccurate pointer decouples from the qubit [27]. Vice versa, a weakly coupled pointer is, necessarily, an inaccurate one.

As in the classical case, to be able to make statistical predictions, Alice needs to make the first measurement accurate, $\Delta f_{1}\to 0$, $G_{1}^{2}\left(f_{1}\right)\to \delta \left(f_{1}\right)$, and pre-select, for example, only those cases where $f_{1}=0$, thereby preparing the system in the state $|I_{0}\rangle$. The rest of the readings are distributed according to (we use $\tilde{\rho }$ to distinguish from the classical distributions of Section 3, Section 4 and Section 5)

(20) $$\begin{array}{cc} {\tilde{\rho }}_{0}\left(f_{2},f_{3},f_{4},f_{5}\right) & =\sum_{l=0,1}{\left|G_{4}\left(f_{4}-l\right)\right|}^{2}\times \\ \; & |\sum_{j=0,1}G_{3}\left(f_{3}-j\right)G_{2}\left(f_{2}-j\right)G_{5}\left(f_{5}+j-1\right)A^{s}\left(F_{l}\leftarrow c_{j}\leftarrow b_{j}\leftarrow I_{0}\right){|}^{2}. \end{array}$$

As in the classical case, Alice can also post-select the qubit, e.g., in a state $|F_{1}\rangle$, by choosing $\Delta f_{4}\to 0$, $G_{4}^{2}\left(f_{4}\right)\to \delta \left(f_{4}\right)$, and collecting the statistics only if $f_{1}=0$ and $f_{4}=1$. The distribution of the remaining three readings is given by

(21) $$\begin{array}{c} {\tilde{\rho }}_{1\leftarrow 0}\left(f_{2},f_{3},f_{5}\right)=W_{1}{\left(t_{4}\right)}^{-1}|\sum_{j=0,1}G_{3}\left(f_{3}-j\right)G_{2}\left(f_{2}-j\right)G_{5}\left(f_{5}+j-1\right)A_{j}^{s}{|}^{2}, \end{array}$$

where we introduce a shorthand

(22) $$\begin{array}{c} A_{j}^{s}\equiv A^{s}\left(F_{1}\leftarrow c_{j}\leftarrow b_{j}\leftarrow I_{0}\right),\;j=0,1. \end{array}$$

The normalisation factor $W_{1}\left(t_{4}\right)\equiv \int \rho_{1\leftarrow 0}\left(f_{2},f_{3},f_{5}\right)$$df_{2}df_{3}df_{5}$ is the probability of reaching the final state $|F_{1}\rangle$ with all three pointers in place, which depends on the pointers’ accuracies

(23) W1(t4)= |A0s|2+|A1s|2+2J2J3J5ReA0s*A1s,Jn≡∫Gn(fn)Gn(fn−1)dfn=exp−12(Δfn)2.

The general rule of the previous section can be extended to the quantum case as follows.

- Alice may ascertain the qubit’s condition, represented by a state in its Hilbert space, *only* when she obtains a pointer’s reading whose probability depends *only* on the system’s path amplitudes for the paths passing through the state in question.

As in the classical case, three accurate measurements allow one to determine the path followed by the qubit. For example, with $\Delta f_{1},\Delta f_{2},\Delta f_{4}\to 0$, outcomes $f_{1}=0$, $f_{2}=1$, and $f_{4}=1$, whose probability is

(24) $$\begin{array}{c} P\left(1,1,0\right)\equiv \int_{-\epsilon }^{\epsilon }df_{2}\int_{1-\epsilon }^{1+\epsilon }df_{4}\int_{-\infty }^{\infty }df_{3}df_{5}\times {\tilde{\rho }}_{0}\left(f_{2},f_{3},f_{4},f_{5}\right)\underset{\Delta f_{2},\;\Delta f_{4}\to 0}{\to }{\left|A_{1}^{s}\right|}^{2}, \end{array}$$

indicates that the qubit has followed the path $\left\{F_{1}\leftarrow c_{1}\leftarrow b_{1}\leftarrow I_{0}\right\}$ (see Figure 4).

## 8. A Quantum “Double-Slit” Problem

The simple model shown in Figure 4 has the essential features of the setup shown in Figure 1 and is simple to analyse. Two paths connect the initial and final states, $|I_{0}\rangle$ at $t=t_{1}$ and $|F_{1}\rangle$ at $t=t_{4}$; pointers $f_{2}$ and $f_{5}$ monitor the presence of the qubit in each path at $t=t_{2}$, and the pointer $f_{3}$ can be used for additional control. For simplicity, Alice can decouple two pointers from the qubit by sending

(25) $$\begin{array}{c} \Delta f_{3},\Delta f_{5}\to \infty ,\;J_{3},J_{5}\to 1. \end{array}$$

As a function of the remaining pointer’s accuracy $\Delta f_{2}$, the distribution of its readings (21) changes from bimodal,

(26) $$\begin{array}{c} {\tilde{\rho }}_{1\leftarrow 0}\left(f_{2}\right)\equiv \int {\tilde{\rho }}_{1\leftarrow 0}\left(f_{2},f_{3},f_{5}\right)df_{3}df_{5}\underset{\Delta f_{2}\to 0}{\to }\frac{|A_{0}^{s}{|}^{2}\delta \left(f_{2}\right)+{\left|A_{1}^{s}\right|}^{2}\delta \left(f_{2}-1\right)}{|A_{0}^{s}{|}^{2}+{\left|A_{1}^{s}\right|}^{2}}, \end{array}$$

to a single broad Gaussian,

(27) ρ˜1←0(f2)=|G2(f2)A0s+G2(f2−1)A1s|2|A0s|2+|A1s|2+2ReA0s*A1s→Δf2→∞G22(f2−z˜),

displaced as a whole by

(28) z˜=ReA1sA0s+A1s,

where we use Equation (A16) of Appendix D. The transformation between the two forms is similar to transformation of the classical probability from (7) to (8) (see Appendix D).

As in the classical case, with an accurate pointer $\Delta f_{2}\ll 1$, a reading is always either 0 or 1, and in every trial, Alice knows the path followed by the qubit.

For a highly inaccurate pointer $\Delta f_{2}\gg 1$, there is not a single reading $f_{2}$ which can be attributed to one path in preference to the other (cf. Appendix C), so Alice never knows *how* the qubit arrived at its final state. Indeed, even the probability of the most likely reading $f_{2}=\tilde{z}$ contains contributions from each path,

(29) $$\begin{array}{c} {\tilde{\rho }}_{1\leftarrow 0}\left(f_{2}=\tilde{z}\right)=\frac{|A_{0}^{s}G_{2}\left(\tilde{z}\right)+A_{1}^{s}G_{2}\left(\tilde{z}-1\right){|}^{2}}{|A_{0}^{s}+A_{1}^{s}{|}^{2}}. \end{array}$$

However, Alice may gain information about a pre- and post-selected ensemble even without knowing the path chosen by the qubit. Having performed many trials (it will take more trials the larger is $\Delta f_{2}$), she can evaluate the average reading, i.e., first moment,

(30) 〈f2〉1←0≡∫f2ρ˜1←0(f2)df2=|A1s|2+J2Re[A0s*A1s]|A0s|2+|A1s|2+2J2Re[A0s*A1s]→Δf2→∞z˜.

There is no contradiction with the Uncertainty Principle, which permits knowing the amplitudes $A_{i}^{s}$, [and, therefore, their particular combination (28)]. What the principle forbids is using this knowledge to answer, among other things, the “which way?” question. We illustrate this with the next example.

## 9. Two Inaccurate Quantum Pointers, and Another Conclusion Not to Make

As in the classical case, Alice can employ at $t=t_{2}$ two highly inaccurate pointers $\Delta f_{2}=\Delta f_{5}\equiv \Delta f\gg 1$, which measure projectors on the states $|b_{0}\rangle$ and $|b_{1}\rangle$, respectively. Now, by Equation (A19), the distribution of the readings is Gaussian,

(31) ρ˜1←0(f2,f5)=|A0sG2(f2)G5(f5−1)+A1sG2(f2−1)G5(f5)|2|A0s|2+|A1s|2+2J2J5ReAs0*A1s→Δf→∞G22(f2−z˜2)G52(f5−z˜5),

where

(32) z˜2=ReA1sA0s+A1s,z˜5=ReA0sA0s+A1s=1−z˜2.

And, as in the classical case, we encourage the reader to avoid the following reasoning (see Section 8):

(i) A pointer “moves” (“weak trace” [6] is produced) [cf. Equation (27)] only when the qubit is in the state upon which the projection is made.
(ii) Equation (31) suggests that both pointers have moved (albeit on average).
(iii) Hence, there is experimental evidence of the qubit’s presence in both states at $t=t_{2}$ and, therefore, in both paths connecting $|I_{0}\rangle$ with $|F_{1}\rangle$.

As in the classical case, we find the fault with using the position of the maximum of the distribution (31). As was shown in the previous section, an inaccurate quantum pointer loses the “which way?” information. The information cannot, therefore, be recovered by employing two, or more, such pointers to predict the presence of the qubit in a given state.

In [29], it was pointed out that assuming that in a double-slit experiment the particle passes through both slits at the same time may lead to a wrong prediction. Namely, only a part of an electron, or photon, would need to be detected at the exit of a slit, and this is not what happens in practice. Next, we briefly review the argument of [29] in the present context.

## 10. A “Wrong Prediction”

If not convinced by the argument of the previous section, Alice can follow the advice of [29], and attempt to study the qubit’s evolution in more detail. In particular, she can add an accurate pointer acting at $t=t_{3}$ (see Figure 4), in order to detect only a part of the qubit travelling along the path $\left\{F_{1}\leftarrow c_{1}\leftarrow b_{1}\leftarrow I_{0}\right\}$. If the distribution (31) is a proof of the qubit being present in both paths at $t_{2}$ in any meaningful sense, this must be the only logical expectation. Since there is no path connecting $|b_{0}\rangle$ with $|c_{1}\rangle$ (see Figure 4), two parts of the qubit cannot recombine in $|c_{1}\rangle$ at $t_{3}>t_{2}$.

However, at $t=t_{3}$, Alice finds either a complete qubit, or no qubit at all. As $\Delta f_{3}\to 0$, the distribution (21) becomes bimodal in a three-dimensional space ($f_{2}$,$f_{3}$,$f_{5}$)

(33) $$\begin{array}{c} {\tilde{\rho }}_{1\leftarrow 0}\left(f_{2},f_{3},f_{5}\right)\underset{\Delta f_{3}\to 0}{\to }\frac{|A_{0}^{s}{|}^{2}G_{2}^{2}\left(f_{2}\right)\delta \left(f_{3}\right)G_{5}^{2}\left(f_{5}-1\right)}{|A_{0}^{s}{|}^{2}+{\left|A_{1}^{s}\right|}^{2}}+\frac{|A_{1}^{s}{|}^{2}G_{2}^{2}\left(f_{2}-1\right)\delta \left(f_{3}-1\right)G_{5}^{2}\left(f_{5}\right)}{|A_{0}^{s}{|}^{2}+{\left|A_{1}^{s}\right|}^{2}}. \end{array}$$

**Figure 5:**
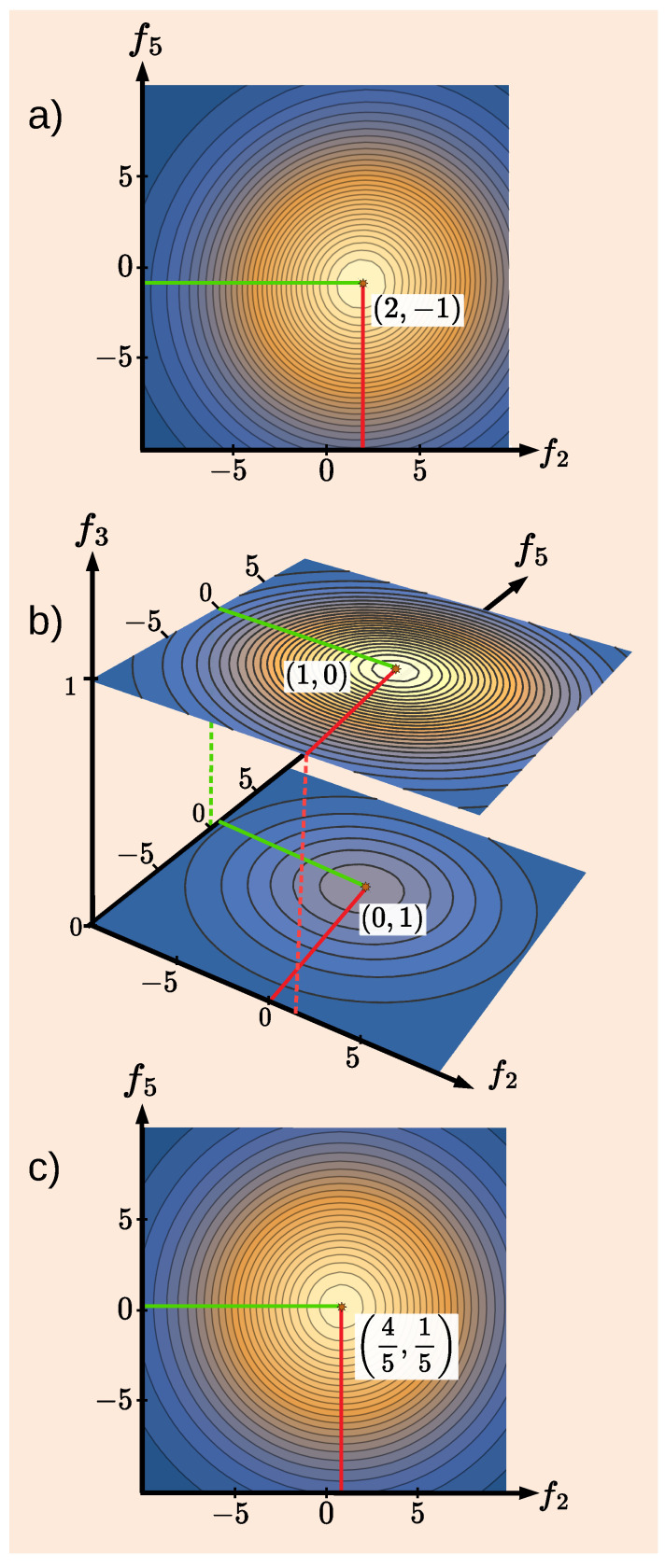
(**a**) Distribution of the readings ${\tilde{\rho }}_{1\leftarrow 0}\left(f_{2},f_{5}\right)$ of two inaccurate quantum pointers with $A_{0}^{s}=-A_{1}^{s}/2$ and $\Delta f_{2}=\Delta f_{5}=10$, monitoring a qubit at $t=t_{2}$ [cf. Equation (31)]; (**b**) The distribution ${\tilde{\rho }}_{1\leftarrow 0}\left(f_{2},f_{3},f_{5}\right)$ in Equation (33) with an accurate pointer, $\Delta f_{3}=0$, added at $t=t_{3}$. (**c**) The result of integrating the distribution in (**b**) over $df_{3}$. Note that in (**c**), one does not recover the distribution shown in (**a**).

Possible values of $f_{3}$ are 0 and 1, and only one of the pointers acting at $t=t_{2}$ is seen to “move” in any given trial. Contour plots of the densities in Equations (31) and (33) are shown in Figure 5a,b, respectively. Note that integrating the density in Figure 5b over $df_{3}$ does not reproduce that in Figure 5a but rather the density (12) [cf. Figure 3a] for a classical system with $P_{0}=1/5$ and $P_{1}=4/5$, shown in Figure 5c.

One can still argue that Alice does not compare like with like since the added accurate pointer perturbs the qubit’s evolution in a way that makes it choose the path $\left\{F_{1}\leftarrow c_{1}\leftarrow b_{1}\leftarrow I_{0}\right\}$. The difficulty with this explanation is well known in the analysis of delayed choice experiments [40]. The decision to couple the accurate pointer may be taken by Alice after $t_{2}$ and cannot be expected to affect the manner in which the qubit passes through the states $|b_{0}\rangle$ and $|b_{1}\rangle$. This argument usually serves as a warning against a naive realistic picture for interpreting quantum phenomena [40]. The conclusion in the case studied here is even simpler. “Weak traces” are not faithful indicators of the system’s presence at a given location, and using them as such leads to avoidable contradictions.

## 11. Quantum “Hidden Variables”

In order to keep the rate of successful post-selections intact, $W_{1}\left(t_{4}\right)={\left|A_{0}^{s}+A_{1}^{s}\right|}^{2}$, Alice may only use weakly coupled and, therefore, inaccurate pointers. These, as was shown above, yield no information as to whether the qubit is in the state $|b_{0}\rangle$ or $|b_{1}\rangle$ at $t=t_{2}$ in any given trial, so the question remains unanswerable in principle. The same is true for classical inaccurate pointers in Section 6, but there it is possible to deduce the probabilities, $P_{0}$ and $P_{1}$, with which the system travels each of the two paths [cf. Equation (15)]. In the quantum case, an attempt to find directly unobservable “hidden” path probabilities governing the statistical behaviour of an unobserved system fails for a simple reason. With no a priori restrictions on the signs of $A_{i}^{s}$, the measured $\tilde{z}$ in Equation (28) can have any real value (see [23]). For a negative $\tilde{z}$, the “probability” ascribed to the path $\left\{F_{1}\leftarrow c_{1}\leftarrow b_{1}\leftarrow I_{0}\right\}$,

(34) $$\begin{array}{c} P_{1}=\tilde{z}W\left(t_{4}\right)<0 \end{array}$$

will also have to be negative. Thus, $P_{1}$ cannot be related to a number of cases in which the system follows the chosen path [41], and a realistic explanation of the double-slit phenomenon fails as expected.

## 12. Summary and Discussion

In summary, a weakly coupled pointer employed to monitor a quantum system is, by necessity, an inaccurate one. As such, it loses information about the path taken by the system in any particular trial, yet one can learn something about path probability amplitudes.

A helpful illustration is offered by a classical case, where a stochastic two-way system is observed by means of a pointer, designed to move only if the system takes a particular path, leading to a chosen destination. The pointer can be rendered inaccurate by making its initial position random. For an accurate pointer, the final distribution of the reading consists of two non-overlapping parts, and one always knows which path the system has travelled. For a highly inaccurate pointer, the final distribution is broad, and not a single reading can be attributed to one path in preference to the other.

The distribution of the initial pointer’s positions can be chosen to be a Gaussian centred at the origin. It is a curious property of broad Gaussians that the final pointer’s reading repeats the shape of the original distribution [cf. Equation (7)], shifted by a distance equal to the probability of travelling the chosen path, conditioned on reaching the desired destination [cf. Equation (8)]. The transition from two maxima to a single peak, achieved when the width of the Gaussian reaches the critical value, is sudden, and can be described as the cusp catastrophe [see Appendix B]. Thus, although the “which way” information is lost in every trial, one is still able to determine parameters (path probabilities) of the relevant statistical ensemble, e.g., by looking for the most probable final reading, or by measuring the first moment of the distribution. For a broad Gaussian, these tasks would require a large number of trials.

The same property of the Gaussians may be responsible for a false impression that two inaccurate pointers [cf. Equation (12) and Figure 3a] move simultaneously (albeit on average), and that this indicates the presence of the system in both paths at the same time. The fallacy is easily exposed by employing one more accurate pointer (see Figure 3b), or simply by recalling that the system cannot be split in two.

Although the quantum case is different, parallels with the classical example can still be drawn. The accuracy of a quantum pointer depends on the uncertainty of its initial position, i.e., on the wave function (19). Weakening the coupling between the pointer and the system has the same effect as broadening the initial state. The distribution of the readings of an accurate pointer consists of two disjoint parts, and one always knows which path has been taken, at the cost of altering the probability of a successful post-selection. The only way to keep the probability intact is to reduce the coupling to (almost) zero, but then there is not a single reading which can be attributed to a particular path.

Owing to the already mentioned property of Gaussians (see Appendix D), the most likely reading of a highly inaccurate pointer is given by the real part of a quantum “weak value” (28), the relative (i.e., normalised to a unit sum) path amplitude. Unlike the classical “weak values” in Equation (9) which must lie between 0 and 1, their quantum counterparts can have values anywhere in the complex plane [23]. As in the classical case, employing a weakly coupled quantum pointer allows one to determine certain parameters (probability amplitudes rather than probabilities) of the quantum ensemble [cf. Equations (15) and (34)].

Equally inadvisable is using the joint statistics of two weak inaccurate quantum pointers [cf. Equation (31)] as evidence of the quantum system’s presence in both pathways at the same time, firstly for reasons similar to those discussed in the classical case and secondly since this would lead to a wrong prediction [29]. An additional accurate pointer always detects either an entire qubit, or no qubit at all, albeit at the price of destroying interference between the paths. In the setup shown in Figure 4, the parts of the qubit, presumably present in both paths, have no means to recombine by the time the accurate measurement is made, hence a contradiction. A similar problem occurs with the interpretation of delayed choice experiments [40], to which we refer the interested reader.

Our concluding remarks can be condensed to few sentences. Unlike the probabilities, the probability amplitudes, used to describe a quantum system, are always available to a theorist. Weak measurements only determine the values of probability amplitudes, or of their combinations. The Uncertainty Principle forbids one to determine the path taken by a quantum system, unless interference between the paths is destroyed [1]. Hence, the weak values have little to contribute towards the resolution of the quantum “which way?” conundrum.

## Data Availability

The study did not report any data.

## Appendix A. Some Properties of Gaussian Distributions

Consider a function

(A1) $$\begin{array}{c} F\left(f\right)=A\exp\left[-\frac{{\left(f-a\right)}^{2}}{{\left(\Delta f\right)}^{2}}\right]+B\exp\left[-\frac{{\left(f-b\right)}^{2}}{{\left(\Delta f\right)}^{2}}\right]\;\; \end{array}$$

with arbitrary real *A*, *B*, *a*, and *b*. For Δf≪|b−a|, *F* has two maxima at f=a and f=b, and a single minimum between them. We are interested in the opposite limit, Δf≫|b−a|, where *F* has a single maximum at

(A2) $$\begin{array}{c} z=\frac{aA+bB}{A+B}, \end{array}$$

easily found by solving $\partial_{f}F\left(f\right)=0$ in the limit Δf→∞. Note that if *A* and *B* have opposite signs, *z* can lie outside the interval [a,b]. In fact, F(f) can be approximated by a single Gaussian

(A3) $$\begin{array}{c} F\left(f\right)\underset{\Delta f\to \infty }{\to }\tilde{F}\left(f\right)=\left(A+B\right)\exp\left[-\frac{{\left(f-z\right)}^{2}}{{\left(\Delta f\right)}^{2}}\right], \end{array}$$

to which it converges point-wise. Indeed, putting x=f/Δf, and expanding the exponentials in Equations (A1) and (A3) in the Taylor series, we find

(A4) $$\begin{array}{c} \left|\frac{F\left(x\right)-\tilde{F}\left(x\right)|}{F(x)}\right|\approx \frac{{\left(b-a\right)}^{2}\left(2x^{2}-1\right)}{{\left(\Delta f\right)}^{2}}\\ \times \left|\frac{AB}{{\left(A+B\right)}^{2}}\right|+o\left({\left(\Delta f\right)}^{-2}\right),\; \end{array}$$

so that the relative error of the approximation (A3) can be made small for any given *f*.

We note further that in the limit Δf→∞, the first moments *F* and $\tilde{F}$ agree [${\langle f^{n}\rangle }_{F}\equiv \int f^{n}F\left(f\right)df/\int F\left(f\right)df$, n=1,2,...]

(A5) $$\begin{array}{c} {\langle f\rangle }_{F}={\langle f\rangle }_{\tilde{F}}=z, \end{array}$$

but the second moments, each of the order of ${\left(\Delta f\right)}^{2}$, differ by a finite quantity,

(A6) $$\begin{array}{c} {\langle f^{2}\rangle }_{F}-{\langle f^{2}\rangle }_{\tilde{F}}=\frac{AB{\left(a-b\right)}^{2}}{A+B}, \end{array}$$

so *F* and $\tilde{F}$ can, at least in principle, be distinguished.

An approximation, similar to the one in Equation (A3), can also be obtained in two dimensions by considering

(A7) $$\begin{array}{c} F\left(\vec{f}\right)=A\exp\left[-\frac{{\left(\vec{f}-\vec{a}\right)}^{2}}{{\left(\Delta f\right)}^{2}}\right]+B\exp\left[-\frac{{\left(\vec{f}-\vec{b}\right)}^{2}}{{\left(\Delta f\right)}^{2}}\right], \end{array}$$

where $\vec{y}=\left(y_{1},y_{2}\right)$ is a two-dimensional vector, and ${\left(\vec{y}\right)}^{2}\equiv y_{1}^{2}+y_{2}^{2}$. As Δf→∞, we find

(A8) $$\begin{array}{c} F\left(\vec{f}\right)\underset{\Delta f\to \infty }{\to }\left(A+B\right)\exp\left[-\frac{{\left(\vec{f}-\vec{z}\right)}^{2}}{{\left(\Delta f\right)}^{2}}\right] \end{array}$$

where

(A9) $$\begin{array}{c} z_{i}=\frac{a_{i}A+b_{i}B}{A+B},\;i=1,2. \end{array}$$

## Appendix B. Connection with Catastrophe Theory

To study the transformation of two maxima and a minimum of the function F(f) in Equation (A1) into a single maximum, we choose a special case a=0 and b=1. The structure of the extrema of F(f) is determined by two parameters R=B/A and Δf, and corresponds, therefore, to cusp the singularity case of the Catastrophe Theory [39]. In the symmetric case R=1, F(f)=F(f+1) and there is always a single extremum at f=1/2. The first and second derivatives at f=1/2 are given by

(A10) $$\begin{array}{cc} \partial_{f}F\left(\frac{1}{2},\Delta f\right) & =0,\\ \partial_{f}^{2}F\left(\frac{1}{2},\Delta f\right) & =-\frac{4e^{-\frac{1}{4{\left(\Delta f\right)}^{2}}}}{{\left(\Delta f\right)}^{4}}\left({\left(\Delta f\right)}^{2}-\frac{1}{2}\right), \end{array}$$

and the three extrema coalesce at $\Delta f=\sqrt{\frac{1}{2}}$, where $\partial_{f}F\left(1/2,\Delta f\right)=0$. This is a case of pitchfork bifurcation [39], shown in Figure A1a. Other cases are shown in Figure A1b,c. Note that with R<0, the single maximum which survives as Δf→∞ lies outside the interval 0≤f≤1.

## Appendix C. The Likelihood of Discovering Which Way a Classical System Went

Consider a probability distribution,

(A11) $$\begin{array}{c} \rho \left(f\right)=\frac{1}{2\left(\Delta f\right)\sqrt{\pi }}\left\{\exp\left[-\frac{f^{2}}{{\left(\Delta f\right)}^{2}}\right]+\exp\left[-\frac{{\left(f-c\right)}^{2}}{{\left(\Delta f\right)}^{2}}\right]\right\},\;\; \end{array}$$

with c>0, and search for a range of *f*, where the first term can be neglected, compared to the second one. For example, one may look for readings where the ratio between the two terms does not exceed some ϵ≪1. Such readings would occur for $f>f_{\epsilon }$, where $f_{\epsilon }={\left(\Delta f\right)}^{2}\left|\ln\epsilon \right|/2c+c/2.$ The probability of finding a reading of this kind is, therefore, $P\left(f>f_{\epsilon }\right)=\int_{f_{\epsilon }}^{\infty }\rho \left(f\right)df$. Replacing $\exp\left[-\frac{f^{2}}{{\left(\Delta f\right)}^{2}}\right]$ by a larger term $\exp\left[-\frac{{\left(f-c\right)}^{2}}{{\left(\Delta f\right)}^{2}}\right]$, we have that [erfc(x) is the complementary error function [42]

(A12) $$\begin{array}{c} P\left(f>f_{\epsilon }\right)<2^{-1}\mathrm{erfc}\left[\frac{f_{\epsilon }-c}{\left(\Delta f\right)}\right]\;\underset{\Delta f\to \infty }{\to }\;\;\frac{c}{2\sqrt{\pi }\left(\Delta f\right)\left|\ln\epsilon \right|}\exp\left[-\frac{{\left(\Delta f\right)}^{2}{\left|\ln\epsilon \right|}^{2}}{4c^{2}}\right]\to 0. \end{array}$$

The probability of finding a value *f*, which can be attributed to only one of the two terms in Equation (A11), vanishes rapidly for (Δf)≫c.

## Appendix D. More Properties of Gaussian Distributions

Consider next a function

(A13) $$\begin{array}{c} F\left(f\right)={\left|A\exp\left[-\frac{{\left(f-a\right)}^{2}}{{\left(\Delta f\right)}^{2}}\right]+B\exp\left[-\frac{{\left(f-b\right)}^{2}}{{\left(\Delta f\right)}^{2}}\right]\right|}^{2} \end{array}$$

with complex valued *A* and *B*, and real *a* and *b*

(A14) $$\begin{array}{c} A=A_{R}+iA_{I},\;B=B_{R}+iB_{I}. \end{array}$$

Equation (A13) can be rewritten as

(A15) $$\begin{array}{c} F\left(f\right)={\left\{A_{R}\exp\left[-\frac{{\left(f-a\right)}^{2}}{{\left(\Delta f\right)}^{2}}\right]+B_{R}\exp\left[-\frac{{\left(f-b\right)}^{2}}{{\left(\Delta f\right)}^{2}}\right]\right\}}^{2}\\ +{\left\{A_{I}\exp\left[-\frac{{\left(f-a\right)}^{2}}{{\left(\Delta f\right)}^{2}}\right]+B_{I}\exp\left[-\frac{{\left(f-b\right)}^{2}}{{\left(\Delta f\right)}^{2}}\right]\right\}}^{2}. \end{array}$$

Applying (A3) to each term in the curly brackets, and then to the sum of the results, yields

(A16) F(f)→Δf→∞F˜(f)=|A+B|2exp−2(f−Re[z])2(Δf)2,

with *z* still given by Equation (A2), but with complex valued *A* and *B*,

(A17) $$\begin{array}{c} z=\frac{a\left(A_{R}+iA_{I}\right)+b\left(B_{R}+iB_{I}\right)}{\left(A_{R}+B_{R}\right)+i\left(A_{I}+B_{I}\right)}. \end{array}$$

An extension to two dimensions can be performed in a similar manner as in Appendix A. For $\vec{f}=\left(f_{1},f_{2}\right)$, $\vec{a}=\left(a_{1},a_{2}\right)$ and $\vec{b}=\left(b_{1},b_{2}\right)$,

(A18) $$\begin{array}{c} F\left(\vec{f}\right)={\left|A\exp\left[-\frac{{\left(\vec{f}-\vec{a}\right)}^{2}}{{\left(\Delta f\right)}^{2}}\right]+B\exp\left[-\frac{{\left(\vec{f}-\vec{b}\right)}^{2}}{{\left(\Delta f\right)}^{2}}\right]\right|}^{2}.\; \end{array}$$

we find

(A19) F(f→)→Δf→∞|A+B|2exp−2(f→−Re[z→])2(Δf)2,

where $\vec{z}$ is still given by Equation (A9) with complex valued *A* and *B*.
